# Anthracyclines-Induced Vascular Endothelial Dysfunction in Cancer Patients and Survivors Using Brachial Flow-Mediated Dilation (FMD) Tool: A Systematic Review and Meta-Analysis

**DOI:** 10.1007/s12012-025-09986-2

**Published:** 2025-04-03

**Authors:** Lana A. Kattan, Sara M. Abulola, Mohamed Izham Mohamed Ibrahim, Zaid H. Maayah

**Affiliations:** 1 Department of Pharmaceutical Sciences, College of Pharmacy, QU Health, Qatar University, 2713 Doha, Qatar; 2 Clinical Pharmacy and Practice Department, College of Pharmacy, QU Health, Qatar University, P.O. Box 2713, Doha, Qatar

**Keywords:** Anthracycline, Endothelial dysfunction, Flow-mediated dilation (FMD), Childhood cancers

## Abstract

Anthracyclines are effective antineoplastic drugs; however, their use is constrained by dose-dependent cardiotoxicity. Vascular endothelial dysfunction is an early independent event in cardiovascular diseases and may precede anthracycline-induced cardiotoxicity. Brachial flow-mediated dilation (FMD) is a non-invasive technique for evaluating vascular endothelial function. We evaluated the evidence on anthracycline-induced vascular endothelial dysfunction in cancer patients and survivors using FMD. Studies measuring FMD in anthracycline-treated active cancer patients or survivors were retrieved from inception to August 2024 using PubMed, Embase, and Scopus. The primary outcome was the difference in FMD between anthracycline-treated patients and healthy controls or baseline. We performed the meta-analysis using a random-effects model and evaluated the certainty in effect estimates. Overall, 18 studies (n = 841 patients) met the inclusion criteria. Compared to the baseline, a non-significant change toward a decline in FMD was observed. However, a significant reduction in FMD was observed in anthracycline-treated patients compared to healthy controls (standardized mean difference (SMD): − 0.6082; 95% CI: − 0.8963 to − 0.3201; p < 0.0001). Subgroup analyses revealed consistent significant reductions in FMD for childhood cancers (SMD: − 0.7189; 95% CI: − 0.9903 to − 0.4476; p < 0.0001), while adult cancers showed no significant difference. No significant publication bias was detected overall for healthy control comparisons. High heterogeneity was observed in the included studies (I^2^ = 81.7808% versus healthy controls and I^2^ = 75.6876% for childhood cancers subgroup analysis). Anthracyclines induce vascular endothelial dysfunction, indicated by lower FMD in cancer patients and survivors, particularly among those with childhood cancers, who might be at risk of long-term cardiovascular complications.

**Supplementary Information:**

The online version contains supplementary material available at 10.1007/s12012-025-09986-2.

## Introduction

Anthracyclines are effective chemotherapies against various malignancies, including breast cancer, lymphomas, and leukemias [1]. However, their clinical utility is limited by dose-dependent cardiotoxicity [1, 2]. Due to advancements in cancer diagnosis and treatment, cancer mortality rates are declining [3]. In long-term survivors, however, cardiovascular complications remain the primary cause of non-cancer-related morbidity and mortality [4]. Compared to the general population, cancer survivors have an eightfold increased risk of cardiovascular-related mortality, which may persist up to 45 years after treatment [4]. Childhood cancer survivors, in particular, often experience a long latency period between their anthracycline treatment and the onset of clinical cardiac dysfunction [5]. Thus, early detection of anthracycline-induced cardiotoxicity can help initiate treatment that protects these populations later in life.

Flow-mediated dilation (FMD), also known as brachial artery reactivity (BAR), is a non-invasive technique for assessing endothelial function by evaluating the peripheral brachial artery endothelium-dependent vasodilation [6]. This technique involves using ultrasound to measure the changes in the brachial artery’s diameter after ischemia (typically for 5 min), which is induced by inflating a blood pressure cuff on the forearm to a level above systolic pressure [7]. FMD prognostic value for cardiovascular events has been demonstrated through several meta-analyses [8–11], which suggests a potential use of this tool in detecting anthracycline-induced endothelial dysfunction. However, despite the established link between endothelial dysfunction and anthracycline-induced cardiotoxicity [12, 13], evidence of the impact of anthracyclines on vascular endothelial dysfunction using FMD remains limited. Therefore, to assess the quality and validity of existing evidence assessing the use of FMD for the measurement of vascular endothelial dysfunction in anthracycline-treated patients, we conducted a systematic review and meta-analysis of studies evaluating the effect of anthracyclines on endothelial dysfunction using FMD. Thus, our goal is to create outcomes from these clinical studies and evaluate the validity of current evidence supporting the effect of anthracyclines on vascular endothelial dysfunction in cancer patients and survivors using FMD.

## Methods

We conducted this systematic review and meta-analysis following the Cochrane Review Protocol [14] and reported it in compliance with the “Preferred Reporting Items for Systematic Reviews and Meta-Analyses (PRISMA)” guideline [15].

### Search Strategy

We performed a comprehensive literature search using PubMed, Embase, and Scopus from inception until August 10, 2024. In addition, we manually screened the reference lists of the included studies to identify any further relevant articles. The search terms were generated based on the “population, intervention, comparison, and outcome (PICO)” question: “In cancer patients and survivors treated with anthracyclines, does anthracyclines induce endothelial dysfunction that could be detected using FMD measurement?”. For the intervention, the term “anthracyclines” along with its variations was utilized, whereas the term “brachial FMD” with its variations was used for the outcome of endothelial dysfunction (Supplementary Table 1).

### Selection Criteria

Results were imported to the Rayyan software program (https://www.rayyan.ai/). Two authors (LK and SA) independently screened the titles and abstracts of identified articles. This was followed by full-text screening which was also done independently. Any discrepancies were addressed through consensus or by consulting a third author (ZM). The authors discussed the reasons for inclusion and exclusion to ensure reliability and consistency in the screening process.

We included peer-reviewed English clinical studies that measured FMD following treatment with any cumulative dose of anthracyclines in patients or survivors of adults or childhood cancers and compared the values with healthy controls or with baseline measurements assessed before receiving anthracyclines. Our exclusion criteria included clinical studies where patients were treated with other therapies that directly affected endothelial function, studies without a baseline measurement or healthy control, duplicate studies, conference abstracts, reviews, book chapters, non-English studies and non-clinical investigations, such as animal studies.

### Data Collection

Data extraction was performed and re-checked by one reviewer (LK) and verified by the other (SA) using a prespecified data extraction tool in Microsoft Excel. Disagreements were resolved by consensus discussions or with the input of a third author (ZM). The data extraction sheet included study characteristics (country of origin and study design), participant demographics (gender, age, sample size, type of cancer, cardiotoxic antineoplastic therapy received, co-interventions if present, and blood pressure), and study outcomes related to FMD (time frame since anthracycline exposure and FMD values).

### Quality Assessment

Two authors (LK and SA) independently performed the quality assessment for all included studies, with any disagreements solved by agreement. The “Joanna Briggs Institute (JBI) critical appraisal checklist” was utilized to evaluate the quality of the studies [16]. Four different JBI checklists were used based on the study design, including the case–control, quasi-experimental, cohort, and analytical cross-sectional checklists. For each question, a study receives a ‘Yes’ if it meets the criteria, a ‘No’ if it does not, and ‘Unclear’ if the information is not clearly mentioned. We used the term ‘Not Applicable’ if the design of the study or the methodology is unrelated to the question. Studies were classified as “low” quality if they failed to meet three or more JBI criteria, “moderate” quality if they did not meet one or two criteria, and “good” quality if they met all JBI criteria. We considered a study to not meet the criteria if it received a ‘No’ or ‘Unclear’ designation for a question.

### Statistical Analysis

Meta-analysis was used to evaluate variations in FMD values in patients treated with anthracyclines compared with healthy controls who did not receive anthracycline treatment. Additionally, it examined the differences in FMD among patients undergoing anthracycline therapy at baseline and post-treatment. FMD was expressed as the mean percent change with standard deviation (SD) of the brachial artery diameter recorded during hyperemia compared to pre-inflation diameter. Since Brouwer et al. 2013, reported the FMD values as median and ranges, we converted the values into the mean and standard deviation using the method reported by Hozo et al. 2005 [17]. Moreover, in Dengel et al. 2008, we combined the mean FMD values for the chemotherapy group and the chemotherapy with the radiation group using a Meta-Analysis Accelerator [18].

We performed meta-analysis using “Jamovi^©^ software version 2.4.11” and we presented the data using a forest plot. Pooled data were used to compute the “standardized mean difference” (SMD) with a “95% confidence interval” using a random-effects model. We used SMD since FMD values were measured differently across studies; thus, standardizing the results was essential to create a uniform scale before combining the results in the meta-analysis. SMD was used to quantify the effect size of each study. We considered the results as statistically significant if *p*-value < 0.05. Heterogeneity was assessed using the Chi-square test and reported as the inconsistency factor (*I*^*2*^*)* value and Cochran’s Q-test. Heterogeneity was considered high if *I*^*2*^ ≥ 50% [14]. Publication bias was assessed by visually inspecting funnel plot asymmetry using “Stata SE software 17.0” and the Egger test [19].

## Results

### Search Results

**Fig. 1:**
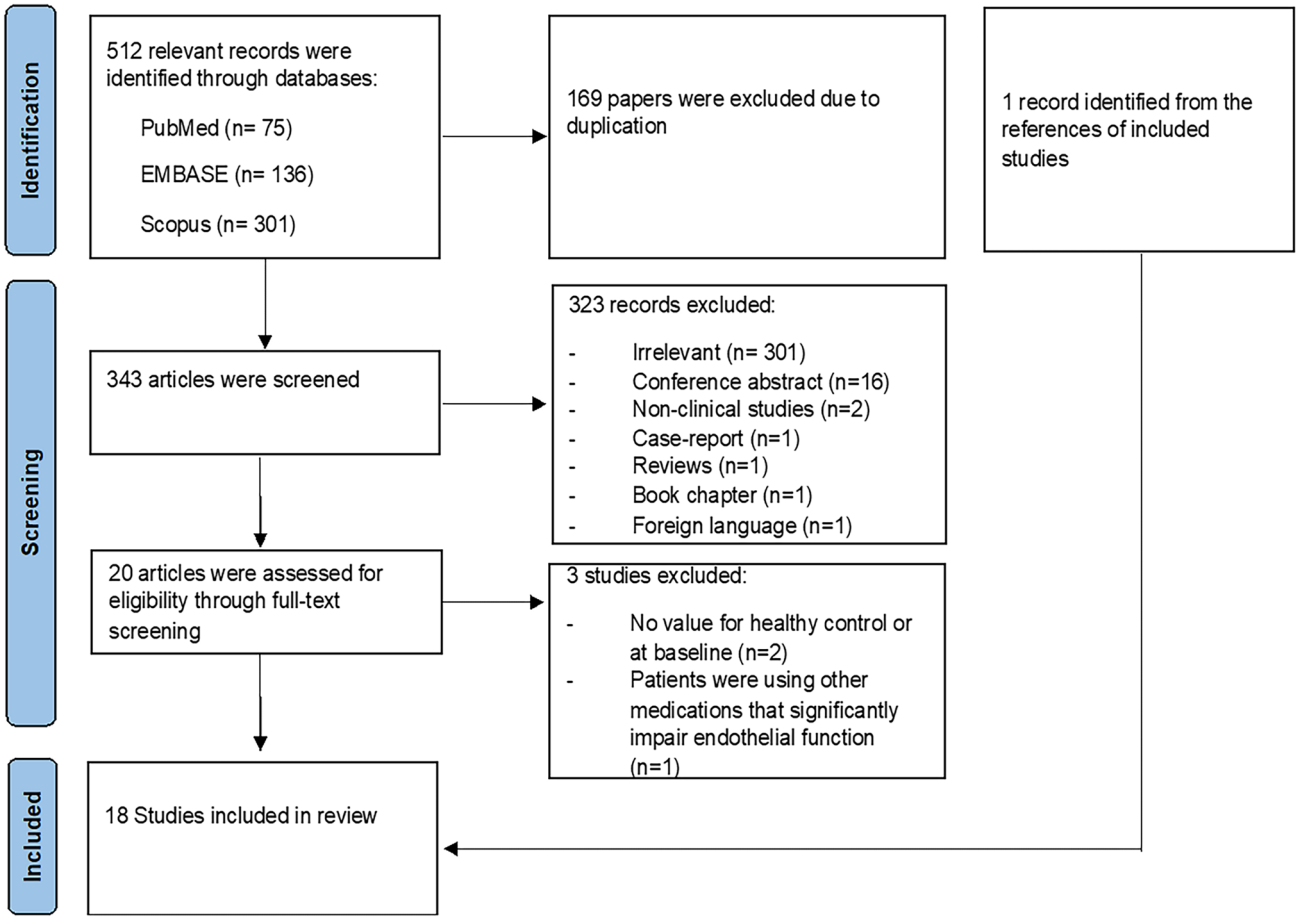
PRISMA flow diagram for studies assessing endothelial function using FMD in anthracycline-treated cancer patients or survivors compared to healthy controls or baseline values

Of 513 relevant records identified from databases and reference screening, 169 records were excluded due to duplications. Based on the title and abstract screening, we excluded 323 records as they were irrelevant, conference abstracts, non-clinical studies, case reports, reviews, book chapters or in a foreign language. This resulted in 20 articles for full-text screening. Two studies were excluded in full-text screening since they did not include FMD values for a healthy control group or at baseline. Moreover, Sutterfield et al. 2018 [20] was excluded as patients were receiving bevacizumab, a vascular endothelial growth factor (VEGF) inhibitor with significant impact on endothelial function and FMD [21]. One study was identified from the references in the included articles. Thus, we included a total of 18 studies in this systematic review and meta-analysis with data evaluating the impact of anthracycline on brachial FMD in cancer patients and survivors (Fig. 1).

### Study Characteristics

The included studies were published between 2001 and 2023. Majority of the studies were conducted in Europe, including Italy (n = 3), Hungary (n = 2), Netherlands (n = 1), Finland (n = 1), Poland (n = 1) and Greece (n = 1), followed by the United States (n = 4), Australia (n = 2), Canada (n = 1), Turkey (n = 1) and Korea (n = 1). Most studies were cross-sectional observational studies (n = 13); other study designs included single cohort studies (n = 3), a case–control study (n = 1) and a quasi-experimental study (n = 1). The main characteristics and outcomes of the included studies [22–39] are summarized in Table 1.

**Table 1:** Characteristics of included studies

Study	Country	Study design	Study population	Cardiotoxic antineoplastic therapy	Sex	Age (years),mean ± SD)	BP for patients (mmHg, mean ± SD)	Outcomes
(Anastasiou et al. 2023)	Greece	Longitudinal case–control study	Breast cancer women (n = 52) and their healthy control women (n = 104)	Anthracycline-based therapy with epirubicin (75 mg/m^2^) plus cyclophosphamide, followed by taxane. 78.8% of patients received trastuzumab, 78.8% received radiotherapy, and 55.8% received endocrine therapy.	100% females	57 ± 12 for cancer patients. 58 ± 10 for healthy controls	SBP post treatment: 127 ± 26 DBP post treatment: 78 ± 10	A significant gradual decrease of FMD in breast cancer patients compared to control from baseline up to one year following the completion of anthracycline treatment, but no difference in FMD from baseline up to 3 months of treatment. The addition of trastuzumab did not cause further decline of FMD. Radiotherapy did not cause any significant changes in FMD.
(Camilli et al. 2023)	Italy	Single-center cross-sectional study	Survivors of childhood cancer, including ALL (n = 8; 40%), lymphomas (n = 6; 30%), Ewing sarcoma (n = 5; 25%), or neuroblastoma (n = 1; 5%) treated with anthracyclines (n = 20) and their matched healthy control (n = 20)	Anthracycline-based therapy with doxorubicin/epirubicin (234.5 ± 87.4 mg/m^2^).	45% females for childhood cancer survivors and 35% females for healthy controls	13.2 ± 2.8 for childhood cancer survivors and12.4 ± 2.9 for control	SBP: 108.0 ± 5.3 DBP: 63.1 ± 2.6	No significant difference in FMD between childhood cancer survivors and controls. FMD was inversely correlated to anthracycline cumulative dose.
(Muggeo et al. 2022)	Italy	Single-center cross-sectional study	Childhood ALL survivors (n = 54) and their matched healthy control (n = 37)	Anthracycline-based therapy. (210 mg/m^2^ in 1 patient, 240 mg/m^2^ in 48 patients (standard and intermediate risk), 310 mg/m^2^ in 1 patient, and 350 mg/m^2^ in 4 patients). Nine patients were exposed to radiotherapy.	35 (65%) females for ALL survivors and healthy controls	9.9 ± 4.2 For ALL survivors. 10.3 ± 2.8 For healthy controls	SBP: 105.6 ± 10.3 DBP: 66.0 ± 7.6	A significantly lower FMD in ALL survivors compared with healthy controls.
(Long et al. 2020)	Australia	Single-center cross-sectional study	Asymptomatic, anthracycline-treated survivors of childhood leukemia (n = 19) and their healthy controls (n = 17)	Anthracycline-based chemotherapy (32% < 100 mg/m^2^; 37% 100–249 mg/m^2^; 32% 250–399 mg/m^2^). 79% of patients received cyclophosphamide and three patients underwent HSCT.	53% females for survivors and controls	19 ± 3 for leukemia survivors. 22 ± 2 for healthy controls	SBP: 111 ± 11 DBP: 63 ± 7	Significantly reduced FMD in leukemia survivors compared to healthy controls.
(Long et al. 2019)	Australia	Single-center cross-sectional study	Long-term childhood leukemia survivors Who received anthracycline (n = 22) and their healthy control (n = 22)	Anthracycline based chemotherapy (41% < 100 mg/m^2^; 27% 100–249 mg/m^2^; 32% 250–399 mg/m^2^). Two patients underwent HSCT.	50% females for survivors and controls	21 ± 3 for leukemia survivors. 25 ± 3 for healthy controls	SBP: 113 ± 11 DBP: 62 ± 6	Significantly impaired FMD in survivors compared to Healthy controls.
(Giordano et al. 2017)	Italy	Single-center cross-sectional study	Childhood ALL survivors (n = 52) and their matched healthy controls (n = 34)	All patients received daunorubicin and doxorubicin. ALL high-risk protocol (n = 6); intermediate risk (n = 29); standard risk (n = 17). 8 patients underwent cranial irradiation.	63% females for ALL survivors. 50% females for controls	9.5 ± 4.1 for ALL survivors. 10.9 ± 4.0 for healthy controls	SBP: 105.5 ± 10.5 DBP: 66.0 ± 7.7	Significantly lower FMD in ALL survivors compared to controls.
(Okur et al. 2016)	Turkey	Single-center cross-sectional study	Children with solid tumors including Hodgkin lymphoma (n = 27; 54%); non-Hodgkin lymphoma (n = 6; 12%); Osteosarcoma (n = 3; 6%); Ewing sarcoma (n = 4; 8%) or others (n = 10; 20%) who were treated with anthracyclines and are in complete remission (n = 50) and their healthy controls (n = 30)	Cumulative doxorubicin dose: Group 1, 38% ≤ 100 mg/m^2^; Group 2, 38% 101–299 mg/m^2^; Group 3, 24% ≥ 300 mg/m^2^ 72% of patients received radiotherapy	30% females for survivors. 40% females for controls	13.5 ± 4.7 for solid cancer survivors. 12.00 ± 4.3 for healthy controls	Not reported	No statistically significant difference in FMD between cancer survivors compared to healthy controls. FMD had a significant negative correlation to the cumulative dose of anthracycline.
(Ederer et al. 2016)	United States	Cross-sectional observational study	Cancer survivors with ≥ 2 years since diagnosis with a treatment history of chemotherapy and/or radiation (n = 8) and their healthy controls (n = 9)	5 patients (63%) had a treatment history of doxorubicin in combination with other chemotherapy agents. 5 patients (63%) had a history of radiotherapy.	88% females for cancer survivors. 78% females for controls	52.4 ± 6.0 for cancer survivors 53.3 ± 4.4 for healthy controls	SBP: 120.9 ± 9.8 DBP: 78.0 ± 6.0	No significant difference in FMD between cancer survivors and controls
(Brouwer et al. 2013)	Netherlands	Single-center cross-sectional study	Adult childhood cancer survivors, including leukemia (n = 113; 41%), lymphoma (n = 56; 20%), sarcoma (n = 48; 17%), brain tumor (n = 32; 12%), and others (n = 28; 10%) treated with potentially cardiovascular toxic anticancer treatment (n = 277) and their healthy sibling controls (n = 130)	199 patients (72%) received anthracycline-based regimens including doxorubicin and daunorubicin. 8% of patients received regimens including platinum (n = 22). 63% of patients underwent radiotherapy.	44% females for cancer survivors. 48% females for controls	Median age for cancer survivors: 27.5 (18.1–48.2) Median age of controls: 25.9 (18.0–51.1)	Median SBP: 114 (78–171) Median DBP: 79 (54–120)	No difference in FMD when comparing childhood cancer survivors and controls.
(Järvelä et al. 2013)	Finland	Quasi-experimental study	Long term childhood ALL survivors (n = 21) and their healthy controls (n = 21)	Total anthracycline dose 120–370 mg/m^2^ (median 240 mg/m^2^)	50% females for survivors and healthy controls	Median age of the survivors: 21.5 (range 16.7–30.3)	Not reported	No significant difference in FMD between survivors and healthy controls at baseline. FMD was significantly lower in male survivors compared to healthy male controls at baseline.
(Jang et al. 2013)	Korea	Single-center cross-sectional study	Children newly diagnosed with high risk ALL who received anthracycline chemotherapy (n = 21) and their healthy controls (n = 20)	Daunomycin in the induction phase and doxorubicin in the delayed intensification phase (142.5 ± 18.2/m^2^)	48% females for anthracyclines treated children. 55% females for controls	10.3 ± 4.3 for anthracyclines treated group 9.6 ± 4.1 for controls	SBP: 111.1 ± 15.6 DBP: not reported	Significantly lower FMD in children with ALL receiving anthracyclines compared to controls. Time since the last anthracycline treatment and age at treatment had no impact on the change in FMD.
(Mizia-Stec et al. 2013)	Poland	Single cohort study	Women with breast cancer scheduled for anthracycline chemotherapy (n = 35)	Epirubicin (n = 16) (414 mg/m^2^) Doxorubicin (n = 15) (278 ± 55 mg/m^2^). 10 patients received radiotherapy. 6 patients received trastuzumab. 18 patients received tamoxifen.	100% females	50 ± 9	SBP post treatment: 135 ± DBP post treatment: 83 ± 9	No significant difference in FMD measured before the first anthracycline dose and six months after the last dose.
(Jenei et al. 2013)	Hungary	Single-center cross-sectional study	Long-term survivors (in complete remission for at least 5 years) of childhood cancers (n = 96) including leukemia (n = 51), lymphoma (n = 12), and others (n = 33) and their matched healthy controls (n = 72)	67 patients received anthracyclines (Doxorubicin, daunorubicin, and idarubicin) as part of combination therapy (< 200 mg/m^2^ (n = 8); 200–250 mg/m^2^ (n = 18); 251–300 mg/m^2^(n = 33); > 300 mg/m^2^ (n = 8)) Patients received dexrazoxane in parallel with anthracycline 21 patients received cranial irradiation	41% females for chemotherapy with anthracycline group 42% females for healthy controls	14.9 ± 5.3 for chemotherapy with anthracycline group. 13.7 ± 4.9 for healthy controls	SBP: 121 ± 3.56 DBP: 81 ± 2.1	Significantly lower FMD in anthracycline treated group compared to the group who received chemotherapy without anthracyclines and compared to healthy controls.
(Dengel et al. 2008)	United States	Cross-sectional study	Young adult survivors of childhood ALL (n = 75) compared to their healthy controls (n = 59)	A total of 42 patients received anthracyclines, including daunorubicin (n = 21), and doxorubicin (n = 21) (1-100mg/m^2^ (n = 10); 101-300mg/m^2^ (n = 11); > 300 mg/m^2^ (n = 8)). 50 patients received radiation.	59% females for cancer survivors 59% females for healthy controls	30.2 ± 7.1 for cancer survivors. 29.62 ± 8.05 for healthy controls	Not reported	Significantly lower FMD in both the chemotherapy and chemotherapy-with-radiation groups compared to healthy controls. No significant difference in FMD between the chemotherapy and chemotherapy-with-radiation group Significantly higher FMD in females compared to males.
(Jones et al. 2007)	Canada	Single Center cross-sectional study	Node-positive and high-risk node- negative patients with operable HER2/neu breast cancer patients (n = 26) and their healthy controls (n = 10)	15 patients (58%) received doxorubicin (60 mg/m^2^) for four cycles. 19 patients (73%) received trastuzumab. 65% received radiotherapy. 62% received tamoxifen.	100% females	48.0 ± 8.5 for cancer patients. 45.2 ± 8.3 years For healthy controls	SBP: 120 ± 14 DBP: 81 ± 9	No significant difference in FMD between HER2 positive breast cancer patients and controls.
(Chow et al. 2006)	United States	Cross-sectional study	Cancer patients who completed anthracycline chemotherapy (n = 14), including patients with Leukemia (n = 7), Ewing sarcoma (n = 3), lymphoma (n = 1), or others (n = 3) pediatric and their healthy controls (n = 14)	Doxorubicin (n = 10) Daunorubicin (n = 4) (> 300 mg/m^2^). Seven patients received radiotherapy.	36% females for cancer patients and controls	14.5 ± 4.54 for cancer patients 11.1 ± 5.11 for healthy controls	SBP: 95.5 ± 5.7 DBP: not reported	Significantly lower FMD in pediatric cancer patients compared to healthy controls. No correlation between the time since anthracycline treatment and FMD.
(Duquaine et al. 2003)	United States	Single cohort study	Breast cancer patients receiving their first dose of doxorubicin (n = 10)	All patients received doxorubicin infusion (60 mg/m^2^)	100% females	52 ± 10	Not reported	Significantly reduced FMD after a single dose of doxorubicin infusion.
(Nagy et al. 2001)	Hungary	Single cohort study	Patients with non-Hodgkin’s lymphoma (n = 18), or Hodgkin’s lymphoma (n = 4) treated with doxorubicin-containing regimen (n = 22)	Doxorubicin (actual dose = 33 ± 12 mg/m^2^) (cumulative dose = 229 ± 112 mg/m^2^) Six patients received mediastinal irradiation. Patients with a greater than 5% lower FMD after the first doxorubicin bolus were pretreated with 1000 mg IV vitamin C before the next doxorubicin dose.	32% females	37.3 ± 13.7	Not reported	Significantly impaired FMD after the first doxorubicin bolus.

*FMD* Flow-Mediated Dilation; *SBP* Systolic Blood Pressure; *DBP* Diastolic Blood Pressure; *ALL* Acute Lymphoblastic Leukemia; *HSCT* Hematopoietic Stem Cell Transplantation

### Patient Characteristics

Overall, 841 patients were covered in this systematic review. Of these, seven studies included active cancer patients [22, 32, 33, 36–39], while 11 studies included cancer survivors [23–31, 34, 35]. For patients with active cancer, FMD measurements were taken as early as 30 min or 11 h after receiving anthracyclines and up to 20 months following the last dose (Tables 2 and 3). Additionally, several studies measured FMD in long-term survivors, ranging from 6 to 25 years post-treatment (Table 3).

**Table 2:** The effect of anthracycline on brachial FMD in clinical studies

Study	FMD (%) before AT Mean ± SD	FMD (%) after AT Mean ± SD	Timeframe
Anastasiou et al. 2023	6.95 ± 2.86 n = 52	5.03 ± 2.83 n = 52	At baseline and 15 months after initiation of treatment
Mizia-Stec et al. 2013	26 ± 15 n = 35	24 ± 11 n = 31	At baseline and 6 months after the last dose of anthracycline
Duquaine et al. 2003	6.5 ± 1.0 n = 10	2.5 ± 1.1 n = 10	At baseline and 27 ± 4 min after a single dose of doxorubicin
Nagy et al. 2001	9.9 ± 4.4 n = 22	6.1 ± 4.6 n = 22	At baseline and 11.2 ± 6.9 h after receiving doxorubicin

**Table 3:** The effect of anthracycline on brachial FMD in clinical studies

Study	FMD (%) for healthy controls Mean ± SD	FMD (%) for cancer patients or survivors Mean ± SD	Timeframe
Anastasiou et al. 2023	6.83 ± 3.22 n = 104	5.03 ± 2.83 n = 52	15 months after initiation of treatment
Camilli et al. 2023	9.41 ± 3.41 n = 20	8.45 ± 1.79 n = 20	6.5 ± 2.7 years since last anthracycline dose
Muggeo et al. 2022	11.6 ± 5.0 n = 37	8.7 ± 3.5 n = 54	At least 3 months since completing chemotherapy
Long et al. 2020	9.65 ± 2.83 n = 17	7.88 ± 1.70 n = 19	12 ± 4 years since final treatment
Long et al. 2019	8.60 ± 1.91 n = 22	6.7 ± 2.1 n = 22	13 ± 4 years since last treatment
Giordano et al. 2017	14.6 ± 4.3 n = 34	9.77 ± 4.44 n = 52	At least 3 months since completing chemotherapy (range: 4–102 months, average 28.2 months)
Okur et al. 2016	8.26 ± 4.56 n = 30	7.38 ± 9.29 n = 50	Mean duration of 36 months in remission (range: 2–204)
Ederer et al. 2016	5.77 ± 2.18 n = 9	10.2 ± 2.48 n = 8	70 months post-treatment
Järvelä et al. 2013	5.78 ± 2.16 n = 21	4.48 ± 2.51 n = 21	A median of 15.9 years (range 11.3–21.4 years) since diagnosis
Brouwer et al. 2013	5.3 ± 6.5 n = 130	5.4 ± 7.92 n = 277	A median of 18.2 years (range: 5.4–30.8) post treatment
Jang et al. 2013	12.1 ± 8.0 n = 20	3.4 ± 3.9 n = 21	2- 85 months since last anthracycline treatment
Jenei et al. 2013	13.13 ± 2.40 n = 72	7.12 ± 6.28 n = 67	Mean of 10.2 years since the last anthracycline dose
Dengel et al. 2008	9.5 ± 2.9 n = 59	6.9 ± 2.6 n = 75	24.6 ± 4.8 years of survival
Jones et al. 2007	8.4 ± 7.7 n = 10	5.4 ± 4.1 n = 26	20 ± 10 months since completing chemotherapy
Chow et al. 2006	6.7 ± 3.3 n = 14	3.8 ± 3.4 n = 14	19.8 ± 18.7 months since last anthracycline dose

Most of the patients had hematologic malignancies, including leukemia and lymphomas. However, some other patients had different types of cancers, such as breast cancer, Ewing sarcoma, and osteosarcoma. Females accounted for 30–100% of patients, with four studies exclusively involving female patients [22, 33, 36, 38]. The doses of anthracyclines varied widely across the studies, with cumulative doses ranging from as low as 75 mg/m^2^ to over 300 mg/m^2^ (Table 1). It was noted that the mean systolic blood pressure (SBP) in the included studies ranged from 95.5 ± 5.7 mmHg to 135 ± 9 mmHg, while mean diastolic blood pressure (DBP) ranged from 62 ± 6 mmHg to 83 ± 9 mmHg. A summary of patient characteristics in the included studies is shown in Table 1.

Six studies included adult cancers [22, 29, 33, 36, 38, 39], while twelve studies focused on childhood cancers [23–28, 30–32, 34, 35, 37] (Table 4). The range of mean age was 37–57 years for adult cancer patients or survivors and 9–30 for childhood cancer patients or survivors. A significant percentage of both adult and childhood cancer patients received concurrent radiotherapy, with variability across studies. However, some studies of childhood cancers excluded patients treated with concurrent radiotherapy [23, 25, 26] (Table 4). Adult cancer patients and survivors showed a higher prevalence of risk factors such as hypertension, dyslipidemia, and diabetes mellitus. Nevertheless, several studies reported excluding patients with diabetes and active smokers [28, 29, 32–34, 37, 38] along with other risk factors for endothelial dysfunction (Table 4). A comparison of the main characteristics of adult and childhood cancer patients and survivors is presented in Table 4.

**Table 4:** Comparison of the Characteristics of Patients and Survivors of Adults and Childhood Cancers in Clinical Studies

Study	Age at FMD measurement (years, mean ± SD)	Age at diagnosis for childhood cancer survivors (years, mean ± SD)	Time since the end of the anthracycline administration	Cumulative anthracycline dose (mg/m^2^)	Concurrent Radiotherapy (Gy)	Risk Factors (metabolic syndrome, obesity, diabetes, hyperlipidemia, hypertension)
*Patients/Survivors of Adult Cancers*						
(Anastasiou et al. 2023)	57 ± 12	N/A	15 months	Epirubicin (300)	Y (78.8%)	Hypertension (35%), Dyslipidemia (37%), Diabetes Mellitus (8%), Active smokers (37%), BMI (kg/m^2^) 26.29 ± 4.40
(Ederer et al. 2016)	52.4 ± 6.0	N/A	70 months	NR	Y (63%)	BMI (kg/m^2^) 25.3 ± 4.0, No diabetes or active smokers
(Mizia-Stec et al. 2013)	50 ± 9	N/A	6 months	Epirubicin (414), Doxorubicin (278 ± 55)	Y (29%)	BMI (kg/m^2^) 26.0 ± 4.8, No diabetes or active smokers
(Jones et al. 2007)	48.0 ± 8.5	N/A	20 ± 10 months	Doxorubicin (240)	Y (65%)	BMI (kg/m^2^) 29 ± 6, Fasting glucose (mmol/L) 4.9 ± 0.5, TC (mmol/L) 5.2 ± 0.9, LDL (mmol/L) 3.1 ± 1.0
(Duquaine et al. 2003)	52 ± 10	N/A	27 ± 4 min	Single dose of doxorubicin (60)	NR	No active smokers, diabetes or hyperlipidemia (total cholesterol > 200 mg/dl)
(Nagy et al. 2001)	37.3 ± 13.7	N/A	11.2 ± 6.9 h	Doxorubicin (229 ± 112)	Y (27%)	Hypertension (50%), Diabetes Mellitus (9%), Obesity (9%)
*Patients/Survivors of Childhood Cancers*						
(Camilli et al. 2023)	13.2 ± 2.8	9.0 ± 11.9	6.5 ± 2.7 years	Doxorubicin (234.5 ± 87.4)	N	Weight (kg) 50.9 ± 15.2
(Muggeo et al. 2022)	9.9 ± 4.2	NR	3 months	(210–350)	Y (17%)	BMI (kg/m^2^) 21.1 ± 4.8, Fasting glucose level (mg/dL) 82.3 ± 6.4, TC (mg/dL) 150.7 ± 23.7, LDL-C (mg/dL) 86.4 ± 18.0
(Long et al. 2020)	19 ± 3	7 ± 5	12 ± 4 years	(< 100–399)	N	Weight (kg) 76.16 ± 19.05
(Long et al. 2019)	21 ± 3	6 ± 4	13 ± 4 years	(< 100–399)	N	Weight (kg) 76.15 ± 19.05
(Giordano et al. 2017)	9.5 ± 4.1	NR	28.2 months (range: 4–102 months)	NR	Y (15%)	BMI (kg/m^2^) 20.9 ± 4.3, Fasting glucose (mg/dL) 82.3 ± 6.4, TC (mg/dL) 150.7 ± 23.1, LDL-C (mg/dL) 86.4 ± 18.0
(Okur et al. 2016)	13.5 ± 4.7	NR	NR	doxorubicin ≤ 100 to ≥ 300)	Y (72%) (mean dose 25.35 Gy; range 10.8–54 Gy)	No active smokers, diabetes, hyperlipidemia, or obesity
(Järvelä et al. 2013)	Median age: 21.5 (range 16.7–30.3)	NR	NR	Total anthracycline dose 120–370 (median 240)	NR	Fasting plasma glucose (mmol/L) 4.98 ± 0.30, Fasting TC (mmol/L) 4.10 ± 1.01, Fasting plasma LDL-C (mmol/L) 2.02 ± 0.97
(Brouwer et al. 2013)	Median age: 27.5 (18.1- 48.2)	Median age at diagnosis: 8.8 (range: 0.0 to 20.1)	18.2 years (range: 5.4–30.8)	NR	Y (63%)	Hypertension (14%), Hypercholesterolemia (33%), Active smokers (25%), BMI (kg/m^2^) median 22.8 (Range 16.4 to 46.3), Fasting glucose (mmol/L) 4.7 (Range 3.1 to 8.7)
(Jang et al. 2013)	10.3 ± 4.3	N/A	2–85 months	(142.5 ± 18.2 mg/m^2^)	Y	No hypertension, hyperlipidemia, diabetes mellitus, or smoke exposure
(Jenei et al. 2013)	14.9 ± 5.3	4.78 ± 2.32	10.2 years	(< 200 to > 300 mg/m^2^)	Y (22%) (dose < 24 Gy)	No active smokers, diabetes mellitus, metabolic syndrome, or impaired glucose tolerance
(Dengel et al. 2008)	30.2 ± 7.1	5.6 ± 4.3	NR	1 to > 300 mg/m^2^	Y (67%) (dose < 25 Gy to ≥ 24 Gy)	BMI (kg/m^2^) 27.45 ± 7.04, TC (mmol/L) 4.77 ± 1.05, LDL-C (mmol/L) 3.22 ± 2.24
(Chow et al. 2006)	14.5 ± 4.54	N/A	19.8 ± 18.7 months	Doxorubicin (350 ± 31.0 mg/m^2^)	Y (50%)	Weight (kg) 52.9 ± 23.4, No diabetes, hypertension, or smoke exposure

*FMD* Flow-Mediated Dilation; *LDL-C* Low-Density Lipoprotein Cholesterol; *TC* Total Cholesterol; *NR* Not Reported; *N/A* Not Applicable, *Y* Yes, *N* No

### Quality Assessment of Studies

**Fig. 2:**
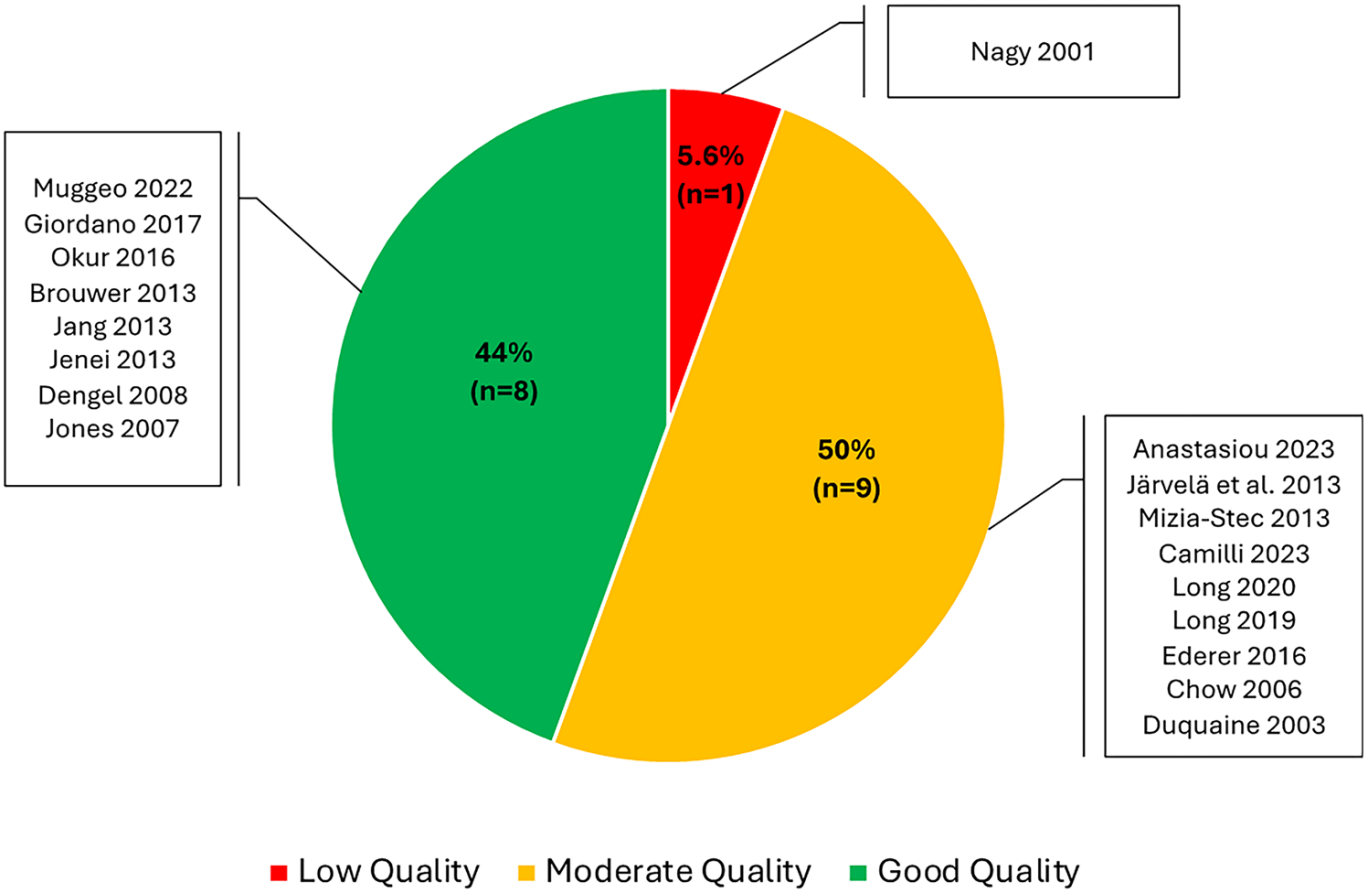
Quality assessment of studies assessing endothelial function using FMD in anthracycline-treated cancer patients or survivors compared to healthy controls or baseline values. The Joanna Briggs Institute (JBI) critical appraisal checklist was used to assess the quality of the studies. Different JBI quality assessment checklists were used according to the study design. Studies were deemed “low” quality, represented by red, if they did not meet three or more JBI appraisal criteria. Studies were deemed “moderate” quality, represented by yellow if they did not meet one or two JBI appraisal criteria and “good” quality, represented by green if they met all JBI criteria.

According to the JBI quality assessment checklist for the 13 cross-sectional observational studies, 8 studies were of high quality (Supplementary Table 5 and Fig. 2). The inclusion criteria, study participants, exposure, outcomes and confounding factors were clearly defined across these studies (Supplementary Tables 2, 3, 4 and 5). Nine studies were of moderate quality, while only one study, Nagy et al. (2001), was considered of low quality due to the lack of strategies to assess confounding factors, the unclear status of participants regarding the outcome of cardiotoxicity at the start of the study, and the insufficient assessment of reasons for loss to follow-up (Supplementary Table 4 and Fig. 2).

### FMD in Anthracycline-treated Patients Compared to Baseline Values Prior to Anthracyclines

**Fig. 3:**
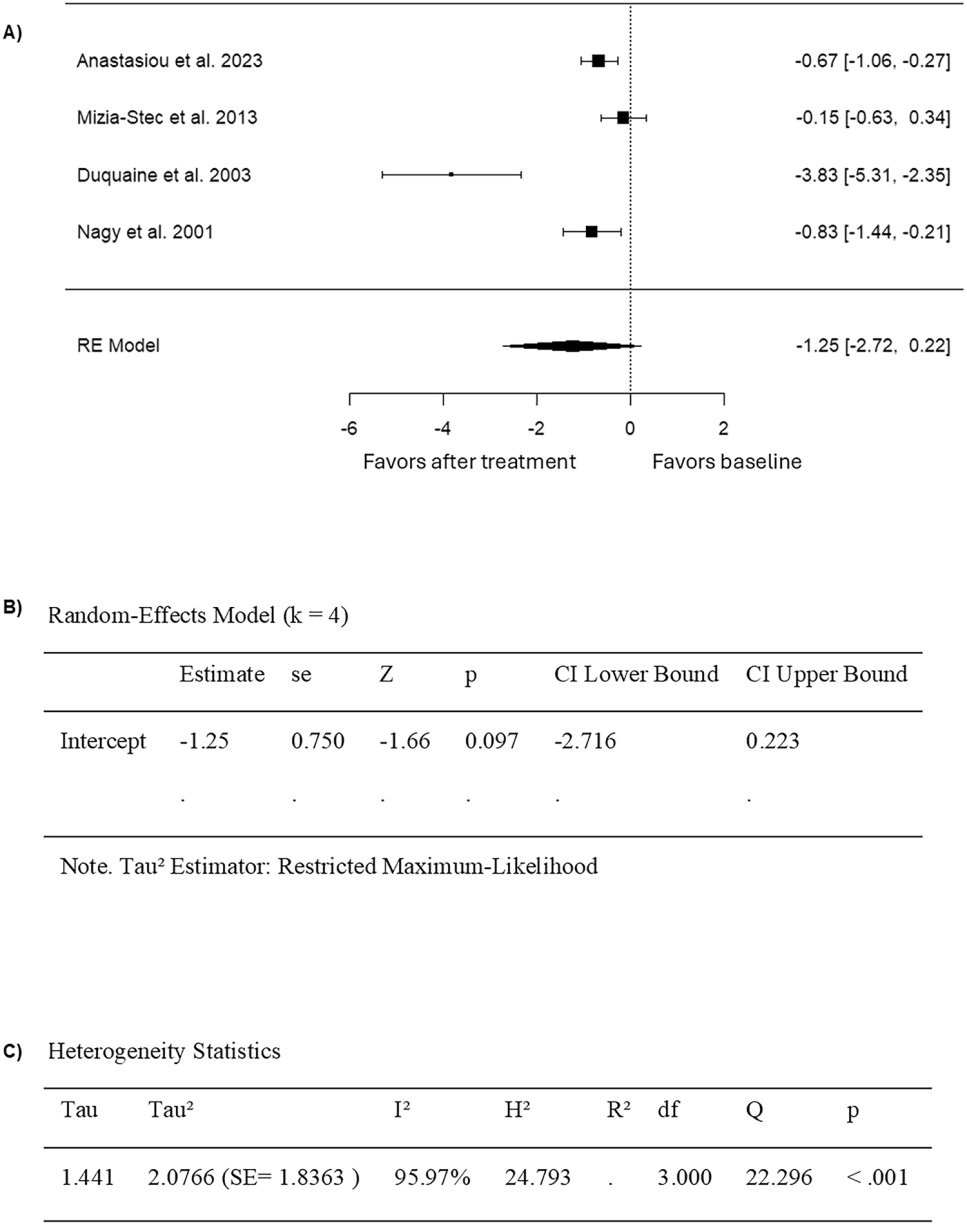
Forest plot of the standardized mean difference in FMD values in anthracycline-treated cancer patients compared to baseline

**Fig. 4:**
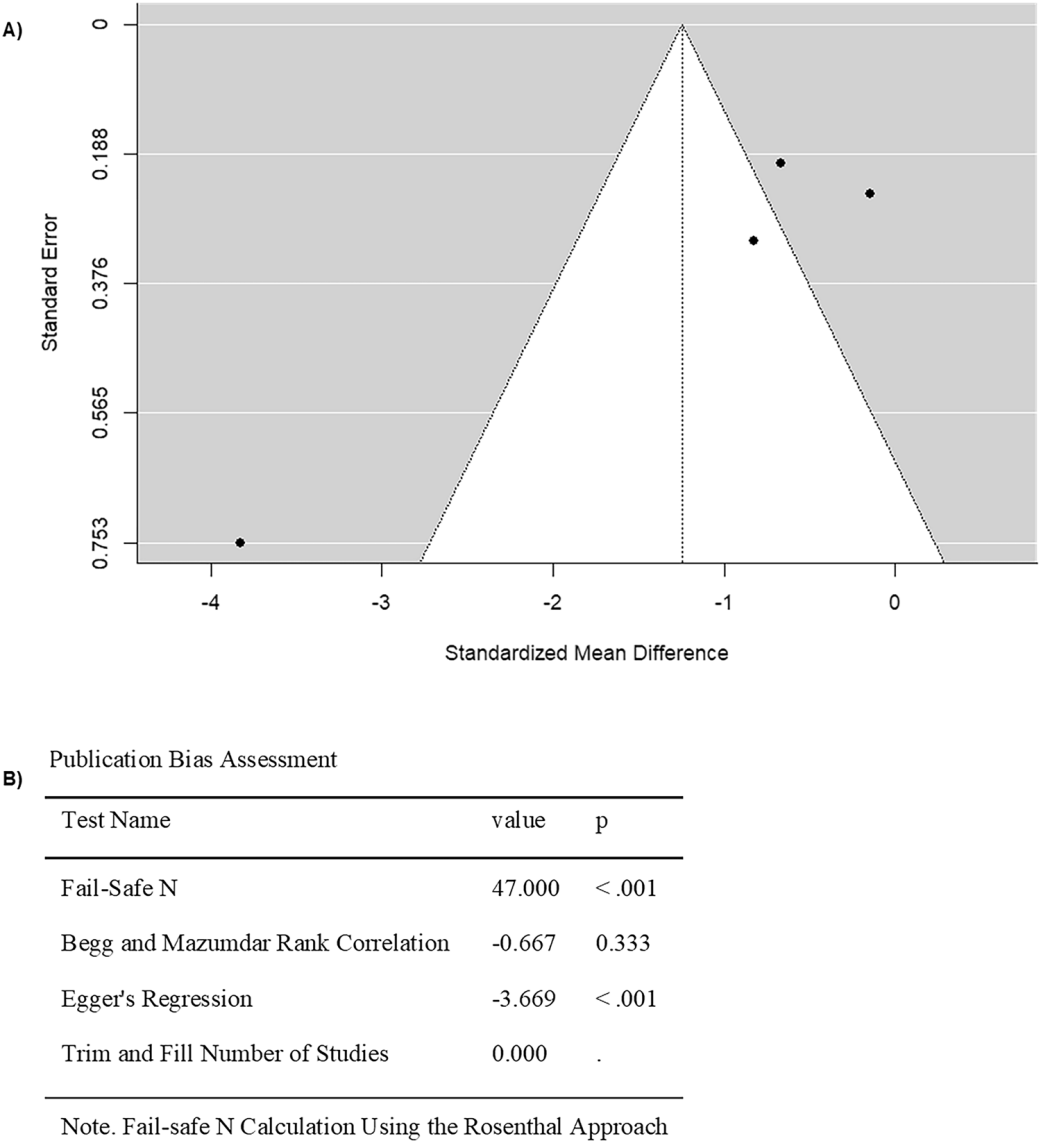
Assessment of publication bias for studies evaluating FMD in anthracycline-treated cancer patients compared to baseline

Four studies that compared FMD values in cancer patients before and after anthracycline chemotherapy were included in the analysis (Table 2). It can be noted that the SMD was from − 3.8306 to − 0.1489, with all results showing a decline in FMD after treatment (Fig. 3). This indicates that endothelial function was consistently worsened after anthracycline treatment across all studies. Using the random-effects model, the average estimated SMD for pooled FMD data was − 1.2464 (95% CI: − 2.7162–0.2234) (Fig. 3). However, the average outcome did not show a significant difference from zero (z = − 1.6620, p = 0.0965) (Fig. 3). Furthermore, while the study by Mizia-Stec et al. (2013) reported FMD values approximately four times higher than those reported by the other studies, excluding this study from the analysis did not change the outcome results. A high heterogeneity was determined across studies according to Cochran’s Q test and I^2^ (Q = 22.2965, p < 0.0001, I^2^ = 95.9665%) (Fig. 3). According to the analysis of the studentized residuals, one study (Duquaine et al. 2003) had a value larger than ± 2.4977 and maybe a potential outlier in this model (Fig. 4). In addition, based on Cook’s distances, none of the studies showed an overly influential effect on the outcome (Fig. 4). Egger’s regression test showed funnel plot asymmetry, whereas the rank correlation test did not (p = 0.0002 and p = 0.3333, respectively), suggesting a potential risk for publication bias (Fig. 4).

### FMD in Anthracycline-treated Patients or Survivors Compared to Healthy Controls

**Fig. 5:**
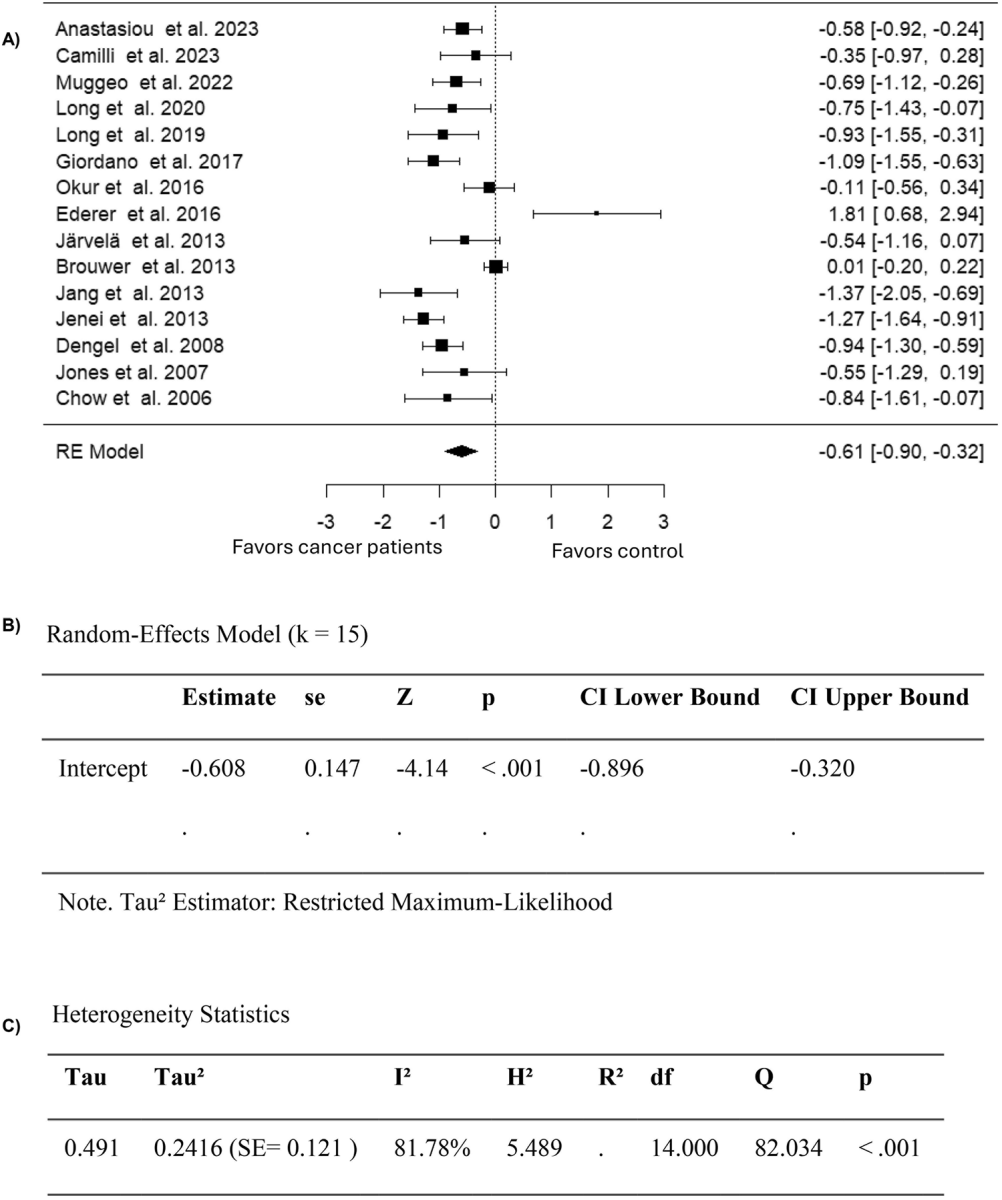
Forest plot of the standardized mean difference in FMD values in anthracycline-treated cancer patients or survivors compared to healthy controls

Among the 18 included studies, 15 studies compared FMD values in cancer patients or survivors to healthy controls (Table 3). The observed SMD in these studies was found to range from − 1.3665 to 1.8084, with 87% of estimates favoring cancer patients or survivors who had undergone treatment with anthracyclines (Fig. 5). Moreover, it can be noted that according to the randoms-effects model, the average estimated SMD for pooled FMD data was − 0.6082 (95% CI: − 0.8963 to − 0.3201) amongst patients who had undergone treatment with anthracyclines (Fig. 5). Consequently, the average outcome exhibited a significant difference from zero (z = − 4.1373, p < 0.0001), confirming the effect of anthracyclines on FMD (Fig. 5). Furthermore, while the study by Ederer et al. (2016) reported higher FMD values for survivors of adult cancers compared to healthy controls, excluding this study from the analysis did not change the outcome results.

**Fig. 6:**
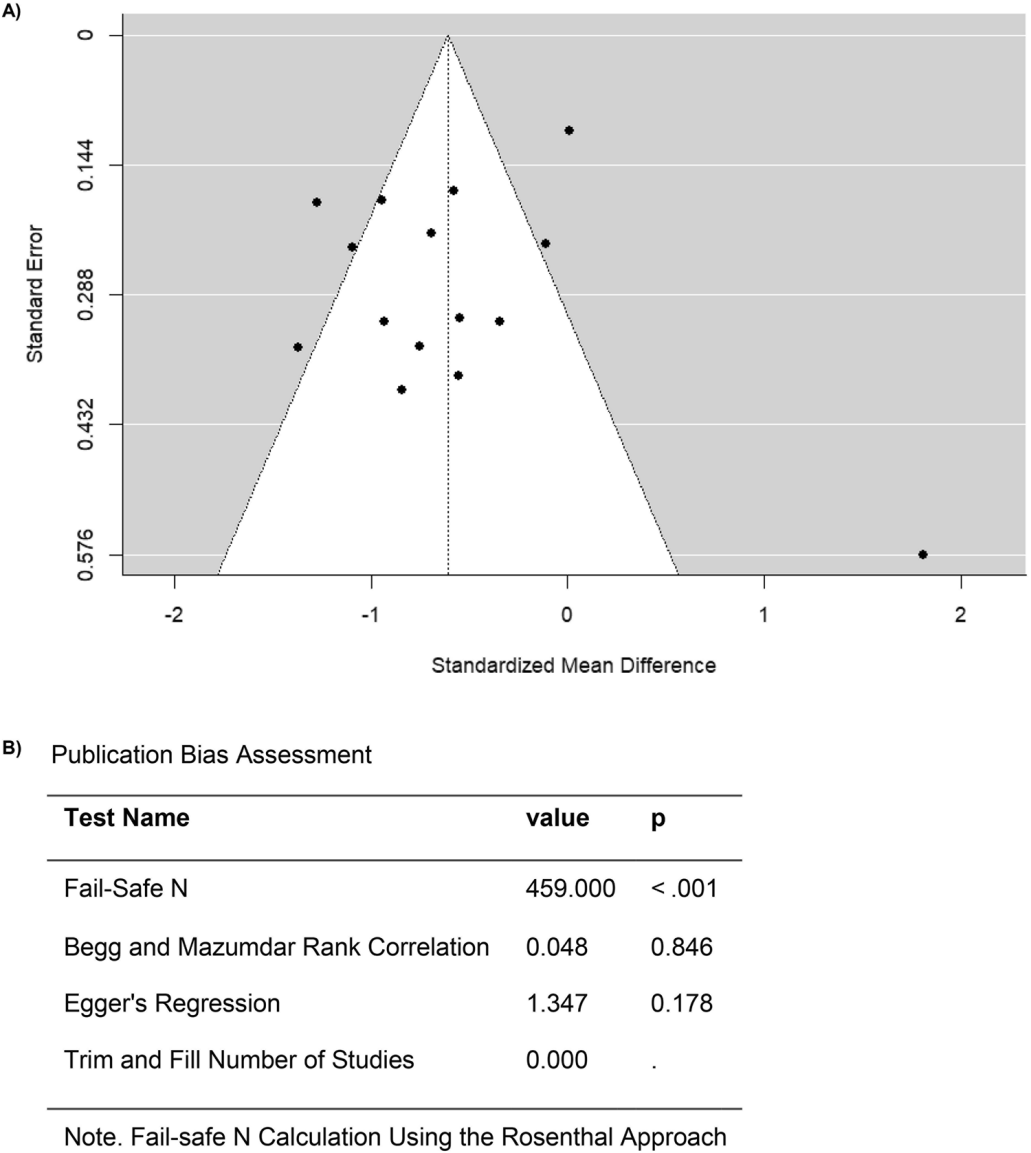
Assessment of publication bias for studies evaluating FMD in anthracycline-treated cancer patients or survivors compared to healthy controls

A high heterogeneity was observed across the studies in the FMD outcome based on the Q-test (Q = 82.0342, p < 0.0001, I^2^ = 81.7808%) (Fig. 5). According to the analysis of the studentized residuals, one study (Ederer et al. 2016) had a value larger than ± 2.9352 and maybe a potential outlier in this model (Fig. 6). Moreover, none of the studies were considered overly influential based on Cook’s distances (Fig. 6). Funnel plot asymmetry was not detected neither by the rank correlation nor by the regression test (p = 0.8458 and p = 0.1779, respectively) (Fig. 6). Overall, these findings confirm that anthracycline-treated cancer patients and survivors showed evidence of endothelial dysfunction, reflected by lower FMD values compared to healthy controls.

### FMD in Anthracycline-treated Patients or Survivors of Adult Cancers Compared to Healthy Controls

**Fig. 7:**
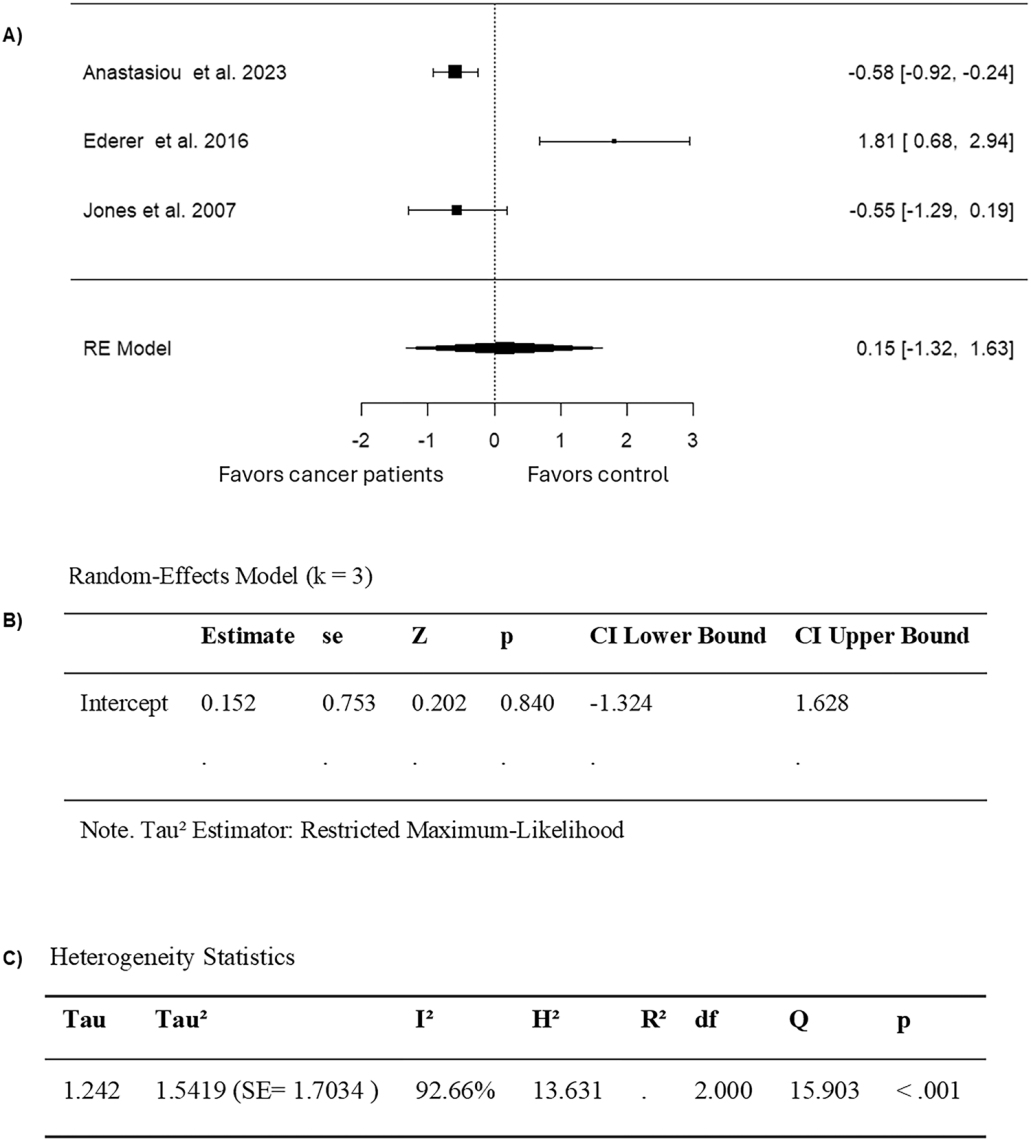
Forest plot of the standardized mean difference in FMD in subgroup analysis of anthracycline-treated adult cancer patients and survivors compared to healthy controls

**Fig. 8:**
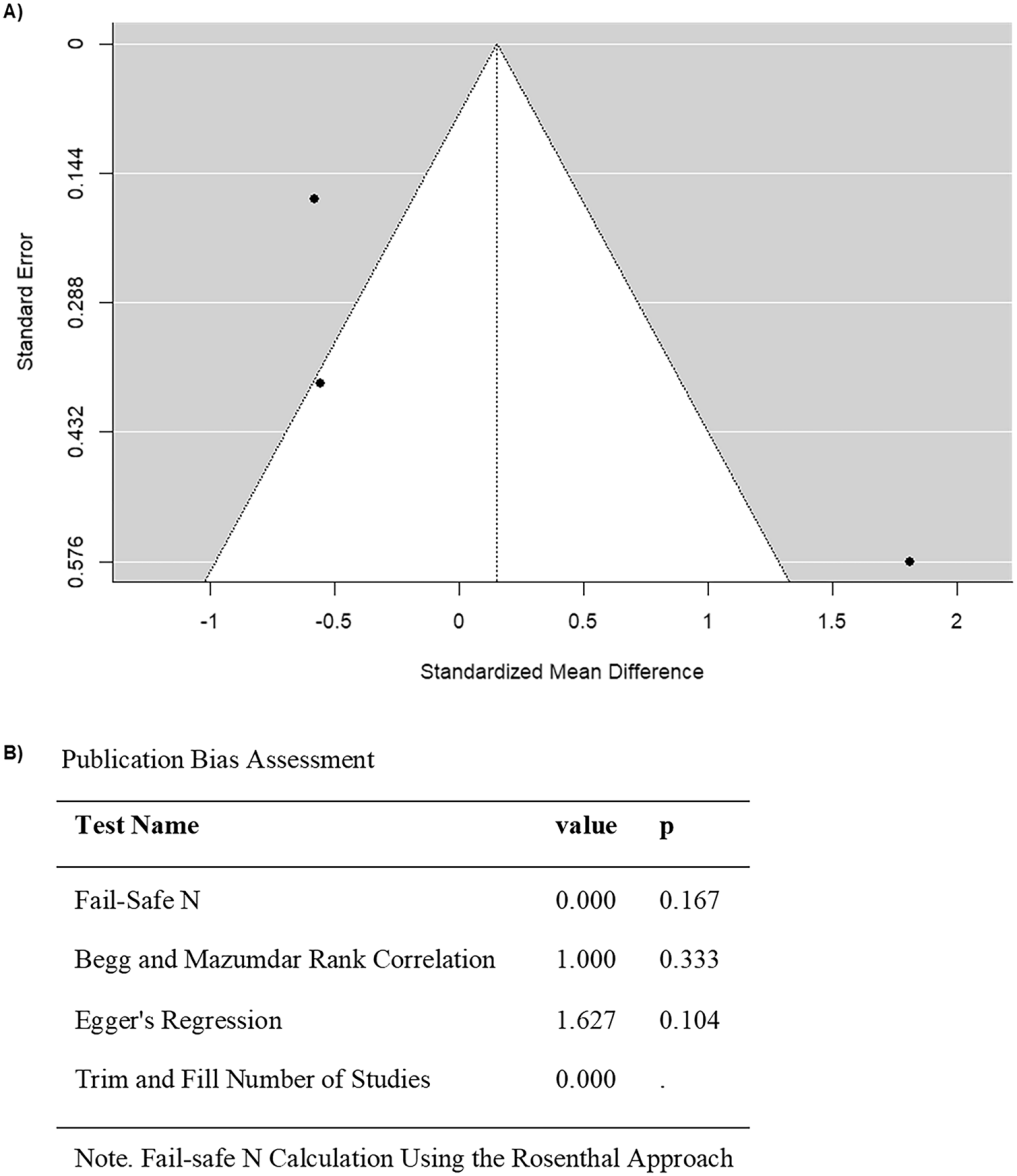
Assessment of publication bias for studies evaluating FMD in subgroup analysis of anthracycline-treated adult cancer patients and survivors compared to healthy controls

When limiting the analysis to studies that only included cancer patients or survivors diagnosed with cancer as adults, 3 studies were included (Table 3). The observed SMD ranged from  −  0.5785 to 1.8084, and 67% of estimates favored adult-onset cancer patients or survivors who received anthracyclines (Fig. 7). Furthermore, according to the random-effects model, the approximate average SMD is 0.1520 (95% CI: − 1.3239 to 1.6280) (Fig. 7). Thus, the average outcome did not exhibit a significant difference from zero (z = 0.2019, p = 0.8400) (Fig. 7). According to the Q-test, high heterogeneity was observed in FMD outcomes among studies that included adult-onset cancers (Q = 15.9034, p = 0.0004, I^2^ = 92.6640%) (Fig. 7). Moreover, after analyzing the studentized residuals, one study (Ederer et al. 2016) was identified as an outlier in this model, with a value greater than ± 2.3940 (Fig. 8). Lastly, there was no overly influential study according to Cook’s distances. (Fig. 8). No funnel plot asymmetry was detected by both the rank correlation and regression test (p = 0.3333 and p = 0.1037, respectively) (Fig. 8). Collectively, these findings suggest that there was no significant effect of anthracyclines on FMD across the majority of adult cancer.

### FMD in Anthracycline-treated Patients or Survivors of Childhood Cancers Compared to Healthy Controls

**Fig. 9:**
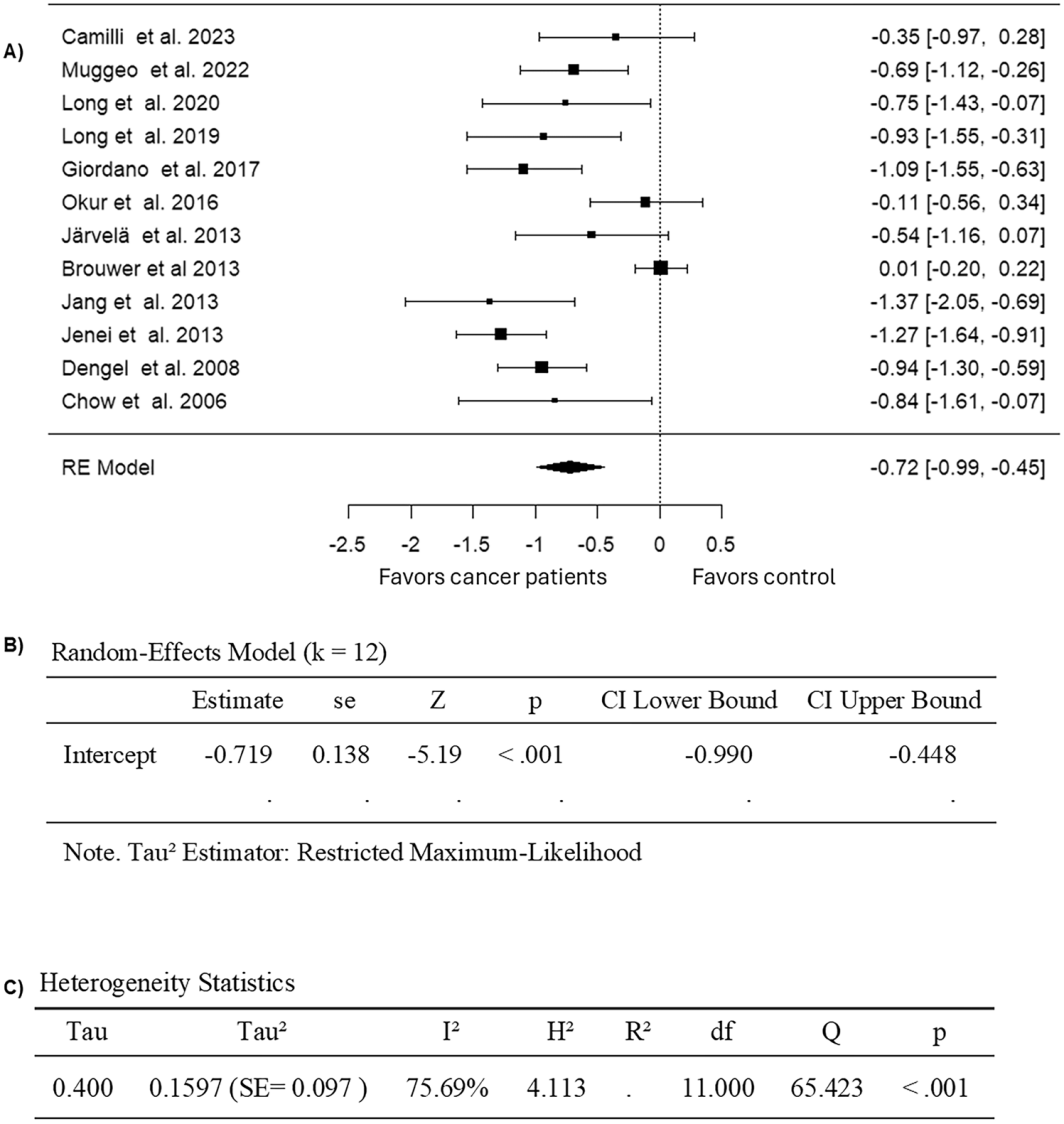
Forest plot of the standardized mean difference in FMD in a subgroup analysis of anthracycline-treated childhood cancer patients and survivors compared to healthy controls

**Fig. 10:**
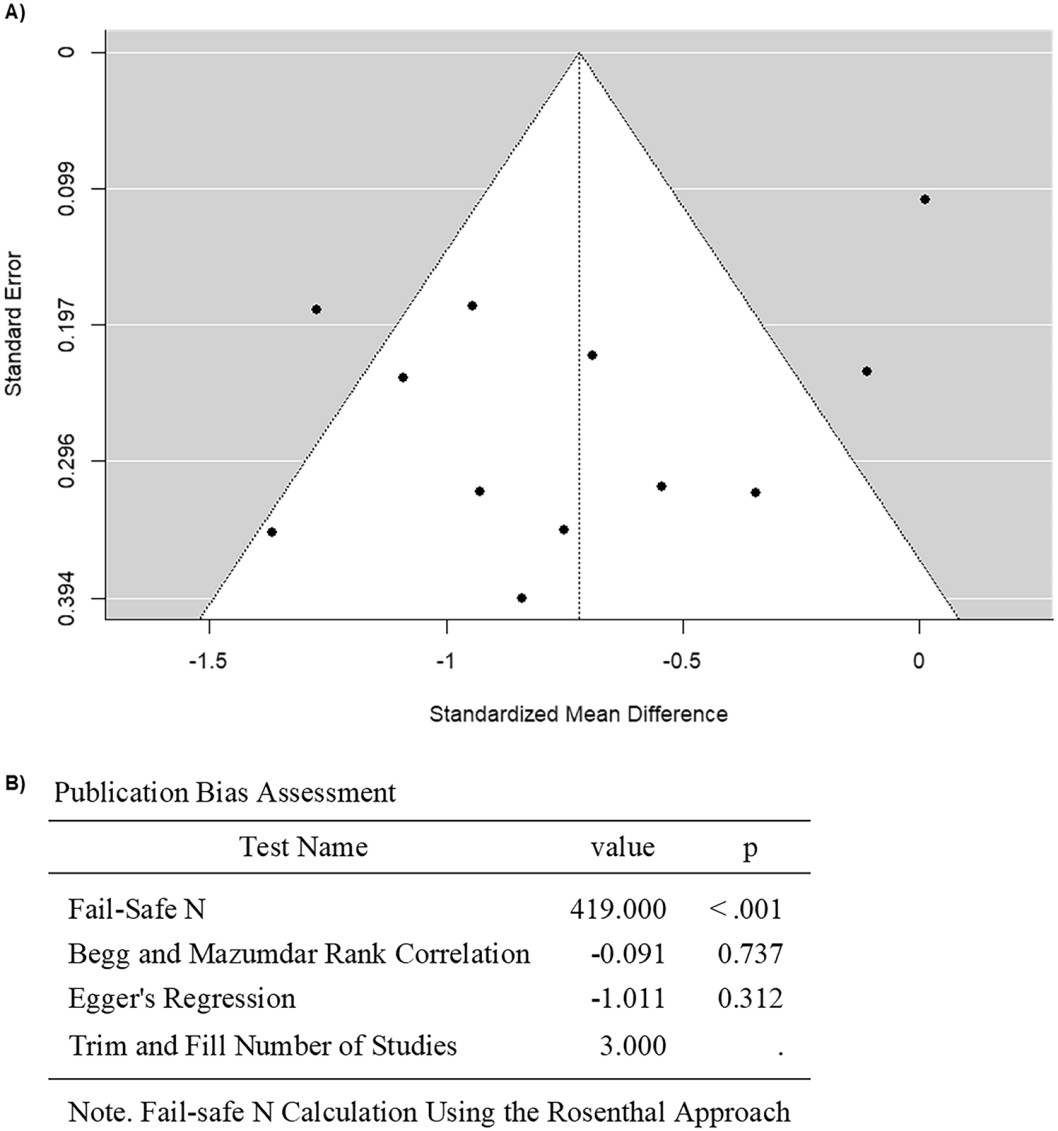
Assessment of publication bias for studies evaluating FMD in subgroup analysis of anthracycline-treated childhood cancer patients and survivors compared to healthy controls

**Fig. 11:**
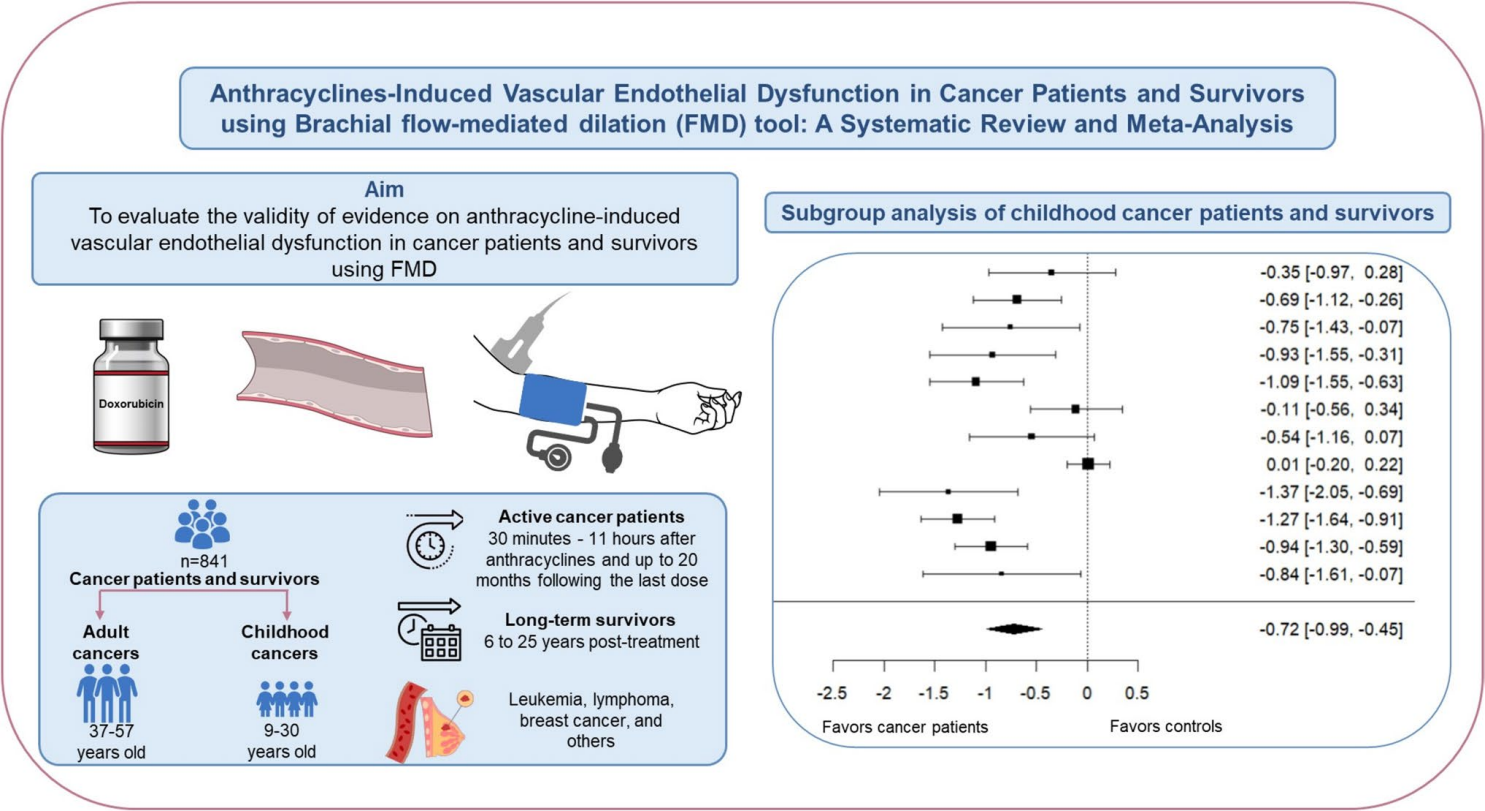
Schematic summary of the study

Upon focusing on studies that included cancer patients or survivors diagnosed with childhood cancers, 12 studies were involved in the subgroup analysis (Table 3). The observed SMD ranged from − 1.3665 to 0.0133, with 92% of estimates favoring childhood cancer patients or survivors treated with anthracyclines (Fig. 9). The estimated average SMD for pooled FMD data from childhood cancer patients or survivors was − 0.7189 (95% CI: − 0.9903 to − 0.4476) based on the random effects model (Fig. 9). Thus, the average outcome exhibited a significant difference from zero (z = − 5.1929, p < 0.0001) (Fig. 9). High heterogeneity in measuring the FMD outcome was observed across the studies as per the Q-test (Q = 65.4228, p < 0.0001, I^2^ = 75.6876%).) (Fig. 9). No outliers were observed in this model since none of the studies had a value greater than ± 2.8653 upon analysis of the studentized residuals (Fig. 10). Moreover, no overly influential study was observed based on Cook's distances (Fig. 10). Lastly, the rank correlation and the regression analysis revealed no funnel plot asymmetry (p = 0.7373 and p = 0.3121, respectively) (Fig. 10). Overall, these findings suggest that treatment with anthracycline is associated with endothelial dysfunction in childhood cancers, which is evident from decreased FMD values (Fig. 11).

## Discussion

Anthracyclines are potent chemotherapeutic agents used to treat a variety of cancers [1]. However, their clinical use is significantly limited by the risk of cardiotoxicity [1, 2]. Given the emerging role of the endothelium in anthracycline-induced cardiotoxicity [12], using reliable tools that assess endothelial dysfunction can help detect subclinical cardiotoxicity, allowing earlier interventions to protect these patients. In humans, endothelial function is assessed by FMD [40], which has demonstrated prognostic value for cardiovascular events [8–11]. Nevertheless, the quality and validity of the existing evidence proving the effect of anthracyclines on endothelial dysfunction using FMD in cancer patients and survivors remain unclear. Therefore, we performed a systematic review and meta-analysis to evaluate the strength of evidence on the impact of anthracyclines on vascular endothelial dysfunction in active cancer patients and survivors, detected using FMD. To our knowledge, this is the first systematic review and meta-analysis that evaluates the validity of FMD as an indicator of anthracycline-induced endothelial dysfunction in active patients and survivors of adult and childhood cancers.

FMD is a marker of the ability of endothelial cells to produce and release nitric oxide (NO) in response to physiological stimuli like increased flow and related shear stress, with lower FMD values indicating impaired NO production or bioavailability, which suggests endothelial dysfunction [41]. Moreover, despite the fact that the brachial artery is a superficial artery, vasodilatory changes in the brachial artery were found to have a reasonable correlation with coronary artery function [41]. Notably, FMD was reduced in anthracycline-treated patients despite having normal blood pressure, as reported in the included studies. This suggests that endothelial dysfunction may precede common cardiovascular risk factors such as hypertension. Consistent with this notion, cancer survivors who received anthracyclines have an increased risk of developing hypertension and coronary artery disease in later stages of life, with pediatric cancer patients, in particular, showing the most significant long-term vascular impacts [42–44].

Our findings showed that subgroup analysis of patients and survivors of childhood cancers revealed a consistent reduction in FMD. These patients often experience a long latency period between treatment with anthracyclines and clinically overt cardiac dysfunction [5]. Moreover, preclinical animal models suggest that anthracyclines induce subclinical cardiotoxicity, which causes molecular changes that increase the heart’s vulnerability to insults occurring later in life despite being clinically silent at younger ages [13, 45, 46]. This highlights the importance of implementing long-term follow-up and screening methods to address the cardiovascular health of childhood cancer patients and survivors. Unlike childhood cancer patients and survivors, while a reduction in FMD was also noted in adult cancer patients following anthracycline treatment compared to their baseline measurements, the difference was not statistically significant. This observation may be attributed to the limited number of studies (n = 4) included in this analysis. Nevertheless, all four studies consistently reported a decline in FMD after anthracycline treatment, suggesting that anthracyclines could also compromise endothelial function in adult cancer patients.

In the present study, we observed a reduction in FMD despite dexrazoxane co-administration in parallel with anthracyclines in some patients [34]. This observation suggests that dexrazoxane may not offer protection against anthracycline-induced vascular endothelial dysfunction. Notwithstanding this information, the protective effects of dexrazoxane on endothelial cells showed mixed results in preclinical animal models. In an acute short-term model of 16 h, dexrazoxane did not protect endothelial cells from anthracycline-induced endothelial dysfunction [47]. On the other hand, recent evidence suggests that in a chronic long-term model of six weeks, pre-treatment with dexrazoxane restored anthracycline-induced endothelial cell dysfunction [48]. However, the mechanism by which dexrazoxane potentially preserves the endothelial function during anthracycline therapy remains yet unexplored [48]. Moreover, longer-term data on the cardioprotective effects of dexrazoxane beyond five years are lacking [5]. Thus, there is a crucial need for novel therapeutic strategies that reduce anthracycline-induced endothelial dysfunction and thus hold the potential to prevent anthracycline-associated cardiotoxicity [12]. In light of this, the European Society of Cardiology (ESC) 2022 guidelines on cardio-oncology recommend heart failure therapy, including angiotensin-converting enzyme inhibitors (ACE-I) or angiotensin receptor blockers (ARB) and beta-blockers for patients who develop symptomatic cancer treatment-related cardiac dysfunction during anthracycline chemotherapy [49]. Moreover, for primary prevention of anthracycline-induced cardiotoxicity in high-risk adult patients, treatment with dexrazoxane, liposomal anthracyclines, ACE-I or ARB, beta-blockers, or statins would be considered [49]. Future studies should focus on approaches that offer long-term cardioprotection against anthracyclines-induced cardiotoxicity, particularly for childhood cancer survivors.

While our findings demonstrated a significant reduction in FMD in cancer patients and survivors, there were some discrepancies in FMD outcomes across a few included studies [29, 31]. For instance, the Ederer et al. study reported higher FMD in survivors of adult cancers compared to healthy controls. This discrepancy might be attributed to the small number of patients recruited in this study, with only five patients with a history of doxorubicin treatment [29]. Unlike Ederer et al.’s study, Brouwer et al.’s study has shown no difference in FMD between cancer patients and healthy controls [31]. This lack of difference might be explained by methodological variations such as differences in cuff occlusion pressure and duration of FMD measurement. Additionally, a single time-point FMD measurement may fail to capture the vascular endothelial changes following cancer treatment [31, 37, 50].

In addition to anthracyclines, it is also important to consider the effect of other anticancer regimens, administered either concurrently or sequentially, on vascular function. Across the included studies, trastuzumab was one of the most common antineoplastic agents used with anthracyclines, as reported in three studies [22, 33, 36]. Notably, trastuzumab is known to cause vascular injury through various mechanisms, however, to a lesser degree than that of anthracyclines [51, 52]. Nevertheless, Anastasiou et al. 2023, showed that the addition of trastuzumab to anthracycline-based chemotherapy did not show a further worsening of endothelial function measured by FMD [22]. Other anticancer regimens with potential effects on vascular function reported in the included studies consist of cyclophosphamide [53, 54], and platinum-based chemotherapy [55], the latter being associated with a reduction in FMD immediately after treatment [56]. Conversely, in two studies, more than half of the recruited patients received tamoxifen [33, 36], which is known to improve endothelial function and increase FMD [57].

Overall, the significant reduction of FMD in anthracycline-exposed patients, particularly those with childhood cancers, highlights the need for regular non-invasive assessment of endothelial function in these patients even years following treatment with anthracyclines. Given that endothelial dysfunction can precede overt cardiotoxicity, FMD can be utilized as a valuable early marker for identifying patients at risk of developing long-term cardiovascular complications. However, it is important to acknowledge the limitations of our study. For instance, high heterogeneity was observed in the included studies, which can be primarily attributed to variations in FMD measurement protocols, measurement conditions, scanning, and analysis. To address this, we used a random-effects model in the analysis. Another important limitation is that FMD protocols vary widely due to differences in testing methods, occlusion cuff placement, cuff occlusion pressure, occlusion duration, and timing of diameter measurements during reactive hyperemia [58]. This emphasizes the need for standardized FMD measurement protocols. Moreover, diagnostic criteria for FMD have not yet been established, which limits the appropriate interpretation and utilization of FMD results in clinical practice. Lastly, while a proposed cutoff value for normal endothelial function assessed by FMD in Japanese patients was 7.1% [58], this value may not be accurate for other races or ethnicities. Thus, multicenter randomized controlled trials are recommended to provide comprehensive data on the clinical utility of FMD for assessing anthracycline-induced endothelial dysfunction and its potential role in early cardiotoxicity detection. As we do not have access to raw patient data, we could not perform a meta-regression on the time from receiving anthracyclines to FMD evaluation or assess the impact of radiotherapy on FMD.

## Conclusion

This systematic review and meta-analysis showed that FMD, a measure of vascular endothelial function, was significantly reduced in anthracycline-treated patients, particularly in childhood cancer patients or survivors, compared to healthy controls, even before any clinical evidence of cardiac dysfunction. These findings suggest that FMD might be a valuable non-invasive tool for the detection of endothelial dysfunction in cancer patients and survivors treated with anthracyclines. In addition, given the role of FMD as a marker of endothelial dysfunction, developing strategies that target anthracycline-induced endothelial damage may help in reducing the risk of anthracycline-induced cardiotoxicity later in life.

## Supplementary Information

Below is the link to the electronic supplementary material.

Supplementary file1 (PDF 165 KB)

## Data Availability

All data used in this systematic review and meta-analysis are available in the original studies cited in the manuscript. A full list of references is provided.
