# Supplementary material for: Anthracyclines-Induced Vascular Endothelial Dysfunction in Cancer Patients and Survivors Using Brachial Flow-Mediated Dilation (FMD) Tool: A Systematic Review and Meta-Analysis
Source: Cardiovasc Toxicol. 2025 Apr 3;25(5):692–718. doi: 10.1007/s12012-025-09986-2 (PMC12018626; doi:10.1007/s12012-025-09986-2)
Supplement: Supplementary file 1 — Supplementary file1 (PDF 165 KB) [file 12012_2025_9986_MOESM1_ESM.pdf]

Supplementary table 1: Full search strategy conducted on Saturday, Aug[10th], 2024

1a. Search strategy used in PubMed

Concepts	Sub-terms	Search options	Number of hits
**1. Anthracyclines**	1.1 anthracyclines	MESH term	80,439 results
	1.2 anthracyclines	All fields	88,689 results
	1.3 doxorubicin	All fields	88,160 results
	1.4 daunorubicin	All fields	76,840 results
	1.5 idarubicin	All fields	2,583 results
	1.6 epirubicin	All fields	8,154 results
	1.7 chemotherapy	Text word	564,678 results
	1.8 cancer-related treatment	TI and AB	101 results
	Total 1= 1.1 OR 1.2 OR 1.3 OR 1.4 OR 1.5 OR 1.6 OR 1.7 OR 1.8		618,929 results
**2. Brachial FMD**	2.1 Brachial artery	MeSH term	10,897 results
	2.2 flow-mediated dilatation	All fields	6,874 results
	2.3 Brachial FMD	All fields	3,692 results
	2.4 Flow-mediated vasodilation	All fields	4,871 results
	Total 2= 2.1 OR 2.2 OR 2.3 OR 2.4		15,986 results
	Grant Total: 1 AND 2		75 results

1b. Search strategy used in Embase

Concepts	Sub-terms	Search options	Number of hits
**1. Anthracyclines**	1.1 anthracycline	ti,ab,kw	22,694 results
	1.2 doxorubicin	ti,ab,kw	78,150 results
	1.3 daunorubicin	ti,ab,kw	8,769 results
	1.4 idarubicin	ti,ab,kw	3,693 results
	1.5 epirubicin	ti,ab,kw	9,446 results
	1.6 chemotherapy	ti,ab,kw	775,768 results
	1.7 cancer-related treatment	ti,ab,kw	32,205 results
	Total 1= 1.1 OR 1.2 OR 1.3 OR 1.4 OR 1.5 OR 1.6 OR 1.7		859,017 results
**2. Brachial FMD**	2.1 Brachial artery	All fields	22,131 results
	2.2 flow-mediated dilatation	All fields	8,077 results
	2.3 Brachial FMD	All fields	6,485 results
	2.4 Flow-mediated vasodilation	All fields	1,904 results
	Total 2= 2.1 OR 2.2 OR 2.3 OR 2.4		26,644 results
	Grant Total: 1 AND 2		136 results

1c. Search strategy used in Scopus

Concepts	Sub-terms	Search options	Number of hits
**1. Anthracyclines**	1.1 anthracycline	TITLE-ABS-KEY	38,534 results
	1.2 doxorubicin	TITLE-ABS-KEY	227,600 results
	1.3 daunorubicin	TITLE-ABS-KEY	32,886 results
	1.4 idarubicin	TITLE-ABS-KEY	11,824 results
	1.5 epirubicin	TITLE-ABS-KEY	32,300 results
	1.6 chemotherapy	TITLE-ABS-KEY	906,953 results
	1.7 cancer-related treatment	TITLE-ABS-KEY	26,063 results
	Total 1= 1.1 OR 1.2 OR 1.3 OR 1.4 OR 1.5 OR 1.6 OR 1.7		1,047,021 results
**2. Brachial FMD**	2.1 Brachial artery	All fields	35,168 results
	2.2 flow-mediated dilatation	All fields	5,698 results
	2.3 Brachial FMD	All fields	4,055 results
	2.4 Flow-mediated vasodilation	All fields	4,444 results
	Total 2= 2.1 OR 2.2 OR 2.3 OR 2.4		38,146 results
	Grant Total: 1 AND 2		301 results

Supplementary table 2: JBI checklist for case-control studies

	Case-Control Checklist	Anastasiou 2023
Q1	Were the groups comparable other than the presence of disease in cases or the absence of disease in controls?	Y
Q2	Were cases and controls matched appropriately?	N
Q3	Were the same criteria used for identification of cases and controls?	Y
Q4	Was exposure measured in a standard, valid and reliable way?	Y
Q5	Was exposure measured in the same way for cases and controls?	Y
Q6	Were confounding factors identified?	Y
Q7	Were strategies to deal with confounding factors stated?	Y
Q8	Were outcomes assessed in a standard, valid and reliable way for cases and controls?	Y
Q9	Was the exposure period of interest long enough to be meaningful?	Y
Q10	Was appropriate statistical analysis used?	Y

Supplementary table 3: JBI checklist for quasi-experimental studies

	**Quasi-Experimental Checklist**	**Järvelä et al. 2013**
Q1	Is it clear in the study what is the 'cause' and what is the 'effect' (i.e. there is no confusion about which variable comes first)?	Y
Q2	Were the participants included in any comparisons similar?	Unclear
Q3	Were the participants included in any comparisons receiving similar treatment/care, other than the exposure or intervention of interest?	N
Q4	Was there a control group?	Y
Q5	Were there multiple measurements of the outcome both pre and post the intervention/exposure?	Y
Q6	Was follow up complete and if not, were differences between groups in terms of their follow up adequately described and analyzed?	Y
Q7	Were the outcomes of participants included in any comparisons measured in the same way?	Y
Q8	Were outcomes measured in a reliable way?	Y
Q9	Was appropriate statistical analysis used?	Y

Supplementary table 4: JBI checklist for prospective cohort studies

	Prospective Cohort Checklist	Mizia-Stec 2013	Nagy 2001	Duquaine 2003
**Q1**	Were the two groups similar and recruited from the same population?	NA	NA	NA
**Q2**	Were the exposures measured similarly to assign people to both exposed and unexposed groups?	NA	NA	NA
**Q3**	Was the exposure measured in a valid and reliable way?	Y	Y	Y
**Q4**	Were confounding factors identified?	Y	Y	Y
**Q5**	Were strategies to deal with confounding factors stated?	Y	N	Y
**Q6**	Were the groups/participants free of the outcome at the start of the study (or at the moment of exposure)?	Y	Unclear	Y
**Q7**	Were the outcomes measured in a valid and reliable way?	Y	Y	Y
**Q8**	Was the follow up time reported and sufficient to be long enough for outcomes to occur?	Y	Y	N
**Q9**	Was follow up complete, and if not, were the reasons to loss to follow up described and explored?	Y	Unclear	Unclear
**Q10**	Were strategies to address incomplete follow up utilized?	N	NA	NA
**Q11**	Was appropriate statistical analysis used?	Y	Y	Y

Supplementary table 5: JBI checklist for analytical cross-sectional studies

	Analytical Cross-sectional checklist	Camilli 2023	Muggeo 2022	Long 2020	Long 2019	Giordano 2017	Okur 2016	Ederer 2016	Brouwer 2013	Jang 2013	Jenei 2013	Dengel 2008	Chow 2006	Jones 2007
**Q1**	Were the criteria for inclusion in the sample clearly defined?	Y	Y	Y	Y	Y	Y	Y	Y	Y	Y	Y	Y	Y
**Q2**	Were the study subjects and the setting described in detail?	Y	Y	Y	Y	Y	Y	N	Y	Y	Y	Y	Y	Y
**Q3**	Was the exposure measured in a valid and reliable way?	Y	Y	Y	Y	Y	Y	Y	Y	Y	Y	Y	Y	Y
**Q4**	Were objective, standard criteria used for measurem	Y	Y	Y	Y	Y	Y	Y	Y	Y	Y	Y	Y	Y

	ent of the condition?													
**Q5**	Were confounding factors identified?	N	Y	N	N	Y	Y	Y	Y	Y	Y	Y	Y	Y
**Q6**	Were strategies to deal with confounding factors stated?	NA	Y	N/A	N/A	Y	Y	N	Y	Y	Y	Y	Y	Y
**Q7**	Were the outcomes measured in a valid and reliable way?	Y	Y	Y	Y	Y	Y	Y	Y	Y	Y	Y	Y	Y
**Q8**	Was appropriate statistical analysis used?	Y	Y	Y	N	Y	Y	Y	Y	Y	Y	Y	N	Y
